# Hypopion inverse

**DOI:** 10.11604/pamj.2014.18.123.4234

**Published:** 2014-06-07

**Authors:** Omar Lezrek, Mounir Lezrek

**Affiliations:** 1 Université Mohammed V Souissi, Service d'Ophtalmologie A de l'hôpital des Spécialités, Centre Hospitalier Universitaire, Rabat, Maroc

**Keywords:** Hypopion inverse, vitrectomie, chambre antérieure, inverse hypopyon, vitrectomy, anterior chamber

## Image en medicine

L'hypopion inverse se voit parfois après une vitrectomie postérieure avec tamponnement par de l'huile de silicone, quand l'huile s’émulsifie, il passe dans la chambre antérieure et s'installe au sommet de la chambre antérieure. Comparer à l'hypopion où les leucocytes se précipitent au fond de la chambre antérieure; et ceci est dû à l'effet de gravité, d'où le nom d'hypopion inverse. Le traitement est basé sur le lavage de la chambre antérieure et aspiration de l'huile de silicone.

**Figure 1:**
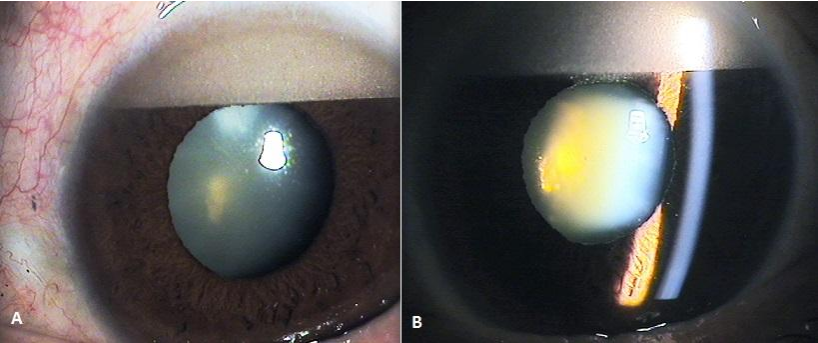
A) Aspect de l'huile de silicone émulsifié dans la chambre antérieure avec un filtre diffuseur de la lumière; B) Aspect de l'huile de silicone émulsifié en hypopion inverse avec une fente fine

